# Structure of an MmyB-Like Regulator from *C. aurantiacus*, Member of a New Transcription Factor Family Linked to Antibiotic Metabolism in Actinomycetes

**DOI:** 10.1371/journal.pone.0041359

**Published:** 2012-07-26

**Authors:** Qingping Xu, Gilles P. van Wezel, Hsiu-Ju Chiu, Lukasz Jaroszewski, Heath E. Klock, Mark W. Knuth, Mitchell D. Miller, Scott A. Lesley, Adam Godzik, Marc-André Elsliger, Ashley M. Deacon, Ian A. Wilson

**Affiliations:** 1 Joint Center for Structural Genomics, La Jolla, California, United States of America; 2 Stanford Synchrotron Radiation Lightsource, SLAC National Accelerator Laboratory, Menlo Park, California, United States of America; 3 Gorlaeus Laboratories, Department of Biology, Leiden University, Leiden, The Netherlands; 4 Center for Research in Biological Systems, University of California San Diego, La Jolla, California, United States of America; 5 Program on Bioinformatics and Systems Biology, Sanford-Burnham Medical Research Institute, La Jolla, California, United States of America; 6 Protein Sciences Department, Genomics Institute of the Novartis Research Foundation, San Diego, California, United States of America; 7 Department of Molecular Biology, The Scripps Research Institute, La Jolla, California, United States of America; Research Center Borstel, Germany

## Abstract

Actinomycetes are important bacterial sources of antibiotics and other secondary metabolites. Many antibiotic gene clusters are controlled by pathway-specific activators that act in response to growth conditions. Here we present the crystal structure of an MmyB-like transcription regulator MltR (PDB code 3pxp) (Caur_2278) from *Chloroflexus aurantiacus*, in complex with a fatty acid (myristic acid). MltR is a distant homolog of the methylenomycin activator MmyB and consists of an Xre-type N-terminal DNA-binding domain and a C-terminal ligand-binding module that is related to the Per-Arnt-Sim (PAS) domain. This structure has enabled identification of a new family of bacterial transcription factors that are distributed predominantly in actinomycetes. Bioinformatics analysis of MltR and other characterized family members suggest that they are likely associated with antibiotic and fatty acid metabolism in actinomycetes. *Streptomyces coelicolor* SCO4944 is a candidate as an ancestral member of the family. Its ortholog in *S. griseus,* SGR_6891, is induced by A-factor, a γ-butyrolactone that controls antibiotic production and development, and is adjacent to the A-factor synthase gen, *afsA*. The location of *mltR*/*mmyB* homologs, in particular those adjacent to less well-studied antibiotic-related genes, makes them interesting genetic markers for identifying new antibiotic genes. A model for signal-triggered DNA-binding by MltR is proposed.

## Introduction

Actinobacteria have attracted significant pharmacological and commercial interest as prolific producers of antibiotics and other secondary metabolites. Genes for antibiotics and other secondary metabolites are typically clustered in the genomes of these bacteria and metabolite production is influenced by a wide variety of environmental and physiological signals [1], [2]. Expression of secondary metabolism genes in actinomycetes is typically subject to multi-level control, which generally involves a specific activator that controls transcription of the pathway, and global control that allows tuning of gene expression in response to growth conditions [3]. The best studied pathway-specific activators are those of the SARP (Streptomyces Antibiotic Regulatory Protein) family, including ActII-ORF4 and RedD in *S. coelicolor* and DnrI in *S. peucetius*, which activate the actinorhodin, undecylprodigiosin and daunorubicin gene clusters, respectively [4]. The global regulators usually contain additional domains for binding small molecules (“signal molecules”) or for interacting with other partners, which serve as a regulatory mechanism for transcription. A well-studied example of the latter is the GntR-family regulator DasR, a pleiotropic regulator of primary and secondary metabolism that controls the onset of development in *Streptomyces* [5]. The global repression of antibiotic production by DasR is relieved by external N-acetylglucosamine, which accumulates as the result of autolytic cell wall degradation and is converted intracellularly to glucosamine-6-P, which acts as an effector molecule [6]. In this way, a rather critical central metabolite controls the production of antibiotics via its interaction with a ligand-binding domain of the transcriptional regulators.

The study of methylenomycin (Mm) biosynthesis in *S. coelicolor* has contributed significantly to the understanding of the genetics of antibiotic production [7], [8]. The genetic elements necessary for the production of Mm and its regulation are contained within the *mmy* gene cluster located on the linear plasmid SCP1. The cluster encodes two biosynthetic systems, regulated by a complex cascade that remains to be fully characterized. Besides the Mm biosynthesis genes, another cluster of genes is required for the production of extracellular 2-alkyl-4-hydroxymethylfuran-3-carboxylic acids that serve as signals for the production of Mm [9]. The Mm synthesis genes are switched on by MmyB [8], [10], which is activated by the aforementioned signal molecules. Furthermore, *mmyB* contains a rare TTA codon and, thus, is subject to translational control by the rare leucyl-tRNA BldA [11]. It has been suggested that MmyB recognizes pseudo-palindromic sequences called “B-boxes” [10]. MmyB contains a likely N-terminal DNA-binding motif that is typical of the Xre family of transcriptional factors. Many homologs of MmyB are found in actinomycetes, suggesting that these proteins may have other functional roles.

We have determined the crystal structure of Caur_2278 from *Chloroflexus aurantiacus* at 2.3 Å resolution with a bound myristic acid, which represents the first structure of an **M**myB-**l**ike **t**ranscription **r**egulator (called MltR hereafter; PDB code 3pxp). *C. aurantiacus* is a Gram-negative, thermophilic, filamentous, phototrophic bacterium regarded as a key model organism for studying the evolution of photosynthesis. MltR functions as a dimer, where each monomer consists of an N-terminal DNA-binding domain with an HTH (helix-turn-helix) motif and a C-terminal PAS-like (Per-Arnt-Sim) domain, which is involved in ligand binding. This structure served as a seed for identifying a large family of transcription factors (>1000 members) found predominately in actinomycetes. MmyB and several other characterized family members from actinomycetes are involved in the production of antibiotics, suggesting a more general role of this newly identified family in the regulation of antibiotic production. We propose a model for MltR-mediated DNA-binding.

## Results and Discussion

### Structure Determination and Model Quality

The crystal structure of MltR was determined using the semi-automated, high-throughput pipeline of the Joint Center for Structural Genomics (JCSG, http://www.jcsg.org), as part of the NIH National Institute of General Medical Sciences (NIGMS), Protein Structure Initiative (PSI) [12], [13]. The selenomethionine derivative of the full length MltR (291 residues) was expressed in *E. coli* with an N-terminal, TEV-cleavable, His-tag and was purified by metal affinity chromatography (see Materials and Methods for details) and the purification tag was removed prior to crystallization. More than 230 crystals were screened for diffraction to identify the best crystal for structure determination. The crystal structure of MltR was determined in space group C2 using the SAD method, and was refined to a resolution of 2.3 Å with an R_cryst_ of 0.168 and an R_free_ of 0.206 (Table 1). The asymmetric unit (asu) contains three monomers (1.5 dimers), which are similar to each other (average rmsd 0.7 Å for 289 C_α_ atoms). Each monomer contains residues 0 to 291 (residue Gly0 is the residue that remains after cleavage of the N-terminal purification tag) and one fatty acid tentatively assigned as myristic acid (MYR). Three ethylene glycols, three chloride ions, and 608 water molecules were also modeled. The final model has good geometry based on MolProbity Ramachandran analysis [14], which shows that all residues are within allowed backbone conformations, with 99% in the most favorable region. The electron density is continuous for the main chain and is also good for the majority of the side chains. Only 1.2% of the side-chain conformations are considered rotamer outliers by MolProbity [14] and 13 surface side chains are partially modeled due to the lack of interpretable electron density.

**Table 1:** Data collection, phasing and refinement statistics (PDB ID 3pxp).

Space group	C2
**Unit Cell**	*a* = 228.8 Å, *b* = 83.6 Å *c* = 54.5 Å, β = 103.1°
**Data collection**	λ_1_ SADSe - peak
Wavelength (Å)	0.9792
Resolution range (Å)	29.7–2.3
No. observations	161,516
No. unique reflections	44,327
Completeness (%)	99.4 (99.6)^a^
Mean I/σ (I)	10.6 (3.1)^a^
R_merge_ on I (%)	8.3 (36.6)^a^
R_meas_ on I (%)	9.7 (43.0)^a^
R_pim_ on I (%)	5.1 (22.3)^a^
Highest resolution shell (Å)	2.42–2.30
**Model and refinement statistics**	
Resolution range (Å)	29.7–2.3
No. reflections (total)	44,326
No. reflections (test)	2,235
Completeness (% total)	99.4
Cutoff criteria	|F|>0
R_cryst_ (%)	16.8
R_free_ (%)	20.6
**Stereochemical parameters**	
Restraints (RMS observed)	
Bond lengths (Å)	0.010
Bond angles (°)	0.93
Average isotropic B-value (Å^2^)	38.4 (37.9, 43.6, 42.8)^b^
ESU based on R_free_ (Å)	0.201
Protein residues/atoms	870/7,213
Molprobity statistics	
All-atom clash score	4.3
Ramachandran plot favored/outlier (%)	99.0/0.00
Side-chain rotamer outliers (%)	1.2

a Highest resolution shell in parentheses.

b B-values for protein, solvent, and ligand are listed in parentheses.

ESU  =  Estimated Standard Uncertainty in atomic coordinates.

R_merge_ = Σ_hkl_Σ_i_|I_i_(hkl)-<I(hkl)>|/Σ_hkl_Σ_i_I_i_(hkl).

R_meas_ (redundancy-independent R_merge_)  = Σ_hkl_[N_hkl_/(N_hkl_-1)]^1/2^Σ_i_|I_i_(hkl)-<I(hkl)>|/Σ_hkl_Σ_i_I_i_(hkl).

R_pim_ (precision-indicating R_merge_) = Σ_hkl_[1/(N_hkl_-1)]^1/2^Σ_i_|I_i_(hkl)-<I(hkl)>|/Σ_hkl_Σ_i_I_i_(hkl).

R_cryst_  =  Σ_hkl_||F_obs_|-|F_calc_||/Σ_hkl_|F_obs_| where F_calc_ and F_obs_ are the calculated and observed structure factor amplitudes, respectively.

R_free_  =  as for R_cryst_, but for 5.0% of the total reflections chosen at random and omitted from refinement.

### Overall Structure of MltR

**Figure 1:**
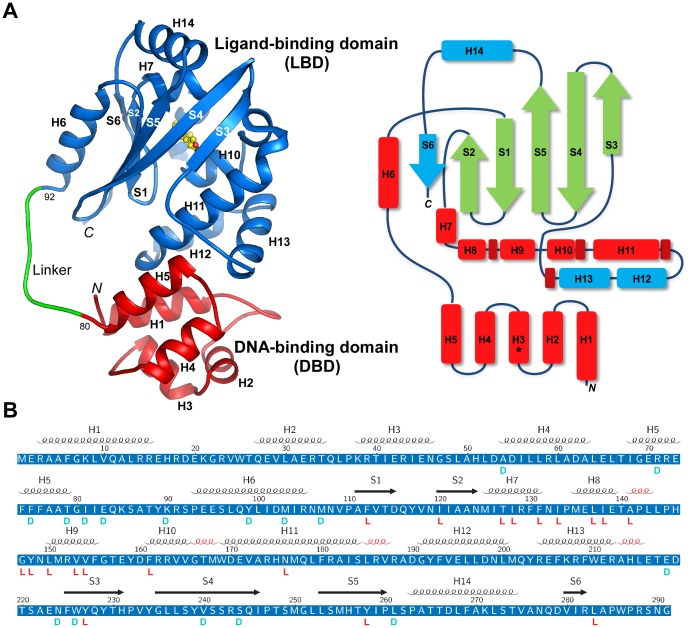
Structure of an MltR monomer. (A) Ribbon diagram of MltR (left). The structure is colored by functional domains; red: DNA-binding (DBD), green: linker, and blue: ligand-binding (LBD). Helices are labeled from H1 to H14, and strands S1 to S6. The myristic acid (MYR) is shown as a ball-and-stick representation with carbons in yellow and oxygens in red. A topology diagram is shown on the right with helices in red and strands in green. The secondary structural elements that are absent in prototypical PAS domains are highlighted in cyan. (B) Protein sequence of MltR annotated with the corresponding secondary structure elements on the top (n.b. the 3_10_ helices are colored red, but not labeled). Residues involved in dimer interface and ligand interactions are marked on the bottom by letters “D” and “L” respectively.

MltR consists of two domains (Fig. 1), a small helical DNA-binding domain (DBD, residues 1-80) and a larger α/β C-terminal ligand-binding domain (LBD, residues 92–291). The DBD contains an HTH motif that is common in many DNA-binding proteins. The C-terminal domain adopts a fold that is related to the PAS domain and binds a putative myristic acid at the expected ligand-binding site. The two domains are connected by a linker (residues 81–91) in an extended conformation. The domain interface is primarily helical, with contributions from H1, H5, H11, H12, H13, and nearby loops (between H4 and H5, H12 and H13, as well as between H13 and S3). Hydrophobic and hydrogen-bonding interactions both contribute to the inter-domain interaction. Leu185 in the C-terminal domain inserts into a hydrophobic pocket formed by Leu9, Leu13, Leu66 and Phe74 in the N-terminal domain, whereas Glu16, Glu70, Arg181, and Arg188 form a hydrogen bond network. The interface between the two domains buries a total surface area of 740 Å^2^.

The modular domain organization of MltR suggests that the protein has evolved from gene fusion between an HTH DNA-binding protein and a ligand-binding protein. This two-domain architecture is common in prokaryotic transcription regulators, and enables the DNA-binding activity to be under the control of an effector molecule.

### MltR Homodimers

**Figure 2:**
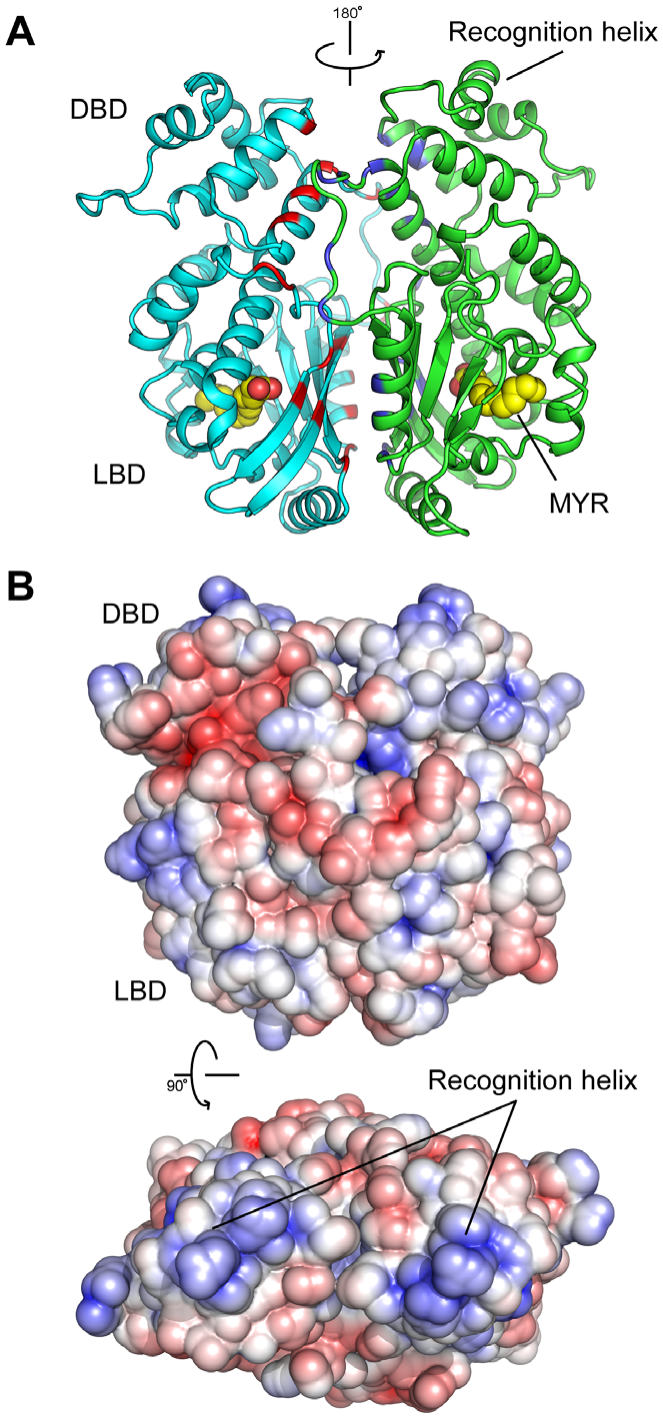
MltR dimer. (A) Ribbon diagram of the MltR dimer with individual protomers colored in cyan and green. Residues within the dimer interfaces are highlighted in red and blue respectively. MYR molecules are shown in spheres. (B) Electrostatic potentials of MltR dimer. The color is scaled from -5 to 5 kT/e (blue, positive; red, negative electrostatic potential).

**Figure 3:**
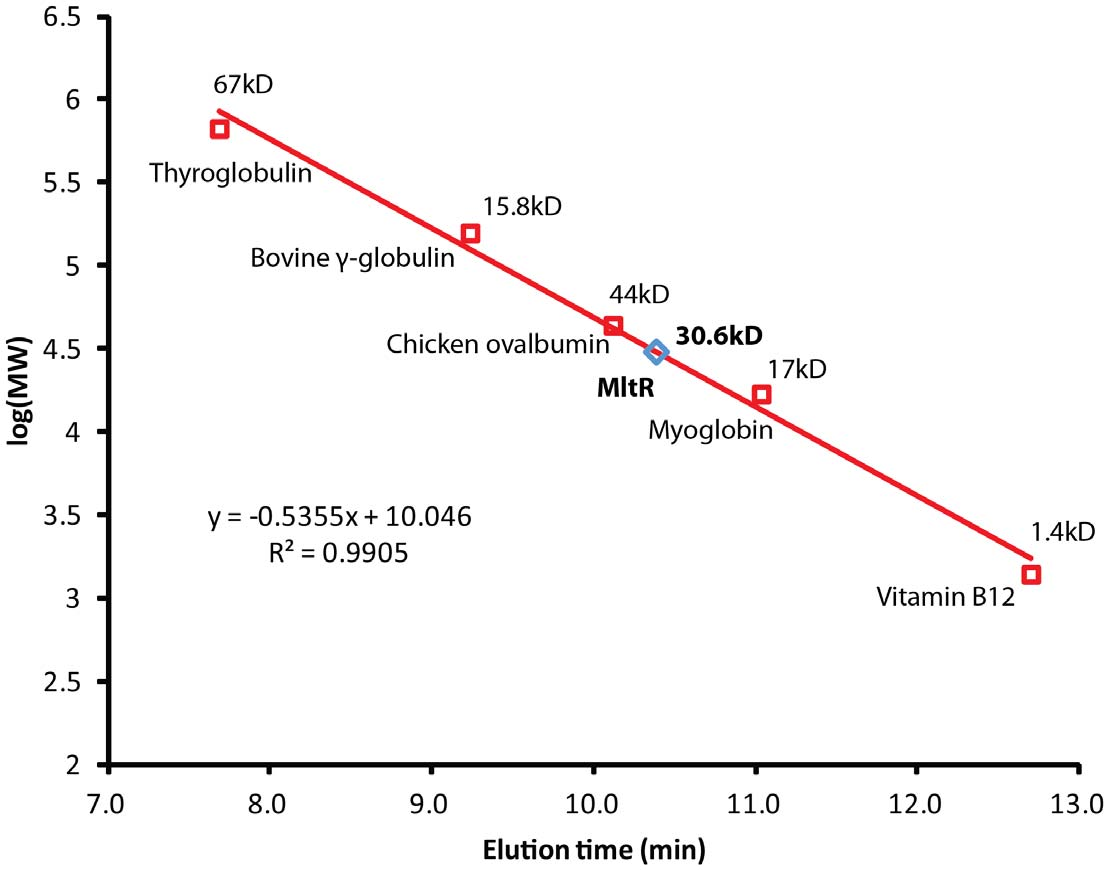
Analytical gel filtration chromatography of MltR. The calibration curve used to estimate the native molecular weight based on the elution position during analytical gel filtration is indicated.

MltR forms two dimers in the crystal lattice. In the first dimer, the two monomers (chains A and B) are related by a non-crystallographic two-fold axis, whereas the second dimer is formed by monomers (chains C and C’) that are related by the crystallographic two-fold axis. However, the two dimers are equivalent to each other (rmsd 0.8 Å for 578 C_α_ atoms). Each dimer is arranged in a side-to-side arrangement with the DBDs at one end and the LBDs at the other, resulting in a relatively flat arrangement with molecular dimension of 75 Å × 73 Å × 48 Å (Fig. 2). However, analytical size exclusion gave an estimated molecular weight of 30.6 kDa (Fig. 3), which corresponds to the calculated molecular weight for a SeMet-MltR monomer (34.5 kD), indicating a monomer as the dominant species in solution. Thus, the dimer is likely induced by crystallization. However, the relative disposition of the DBDs in the dimer is similar to members of the Xre family, suggesting the dimer is, indeed, physiologically relevant (see below).

The dimer buries a total surface area of ∼3100 Å^2^ (∼1550 Å^2^ per monomer) with a gap index of 3.8, which agree with statistical averages of known homodimers [15]. Both domains, as well as the linker region, contribute to the dimer interface, primarily through hydrophobic interactions. The contribution from the DBDs involves helices H4 and H5 and the linker region. Ile83 (of one monomer) interacts with Ala54′, Leu57′, Leu8′, and Phe75′ (of the other subunit). Furthermore, Glu83 forms two hydrogen bonds with Arg71′. The contribution from the LBDs is mediated through two hydrophobic patches on the surfaces of H6 (Tyr99, Met103, Met107) and the β-sheet (Trp226′, Val240′, and Leu261′), and one hydrogen bond between Asn224 and Ser244′. Furthermore, the linker region also is in contact with the LBD and makes significant contribution to the dimer interface.

### DNA-binding Domain

The DBD consists of five helices (H1–H5). The HTH motif comprises of helices H2 and H3, which are short helices (∼10 aa) connected by a three-residue turn. Sequence analysis clearly indicates that the N-terminal DNA binding domain belongs to the Xre family (or HTH type 3 family in PFAM notation) of transcription factors, which itself is a member of a huge superfamily of HTH motif proteins [*NB* This family was named after the *Bacillus subtilis* prophage PBSX encoded repressor Xre [16], and not to be confused with xenobiotic response element (XRE), which is also involved in DNA binding]. The Xre family contains more than 35,000 proteins and more than 70 structures are available, including the well-studied phage Cro repressor and other regulators of diverse functions. Structures of the Xre family HTH modules are highly conserved with five helices assembled into a small globular domain.

**Figure 4:**
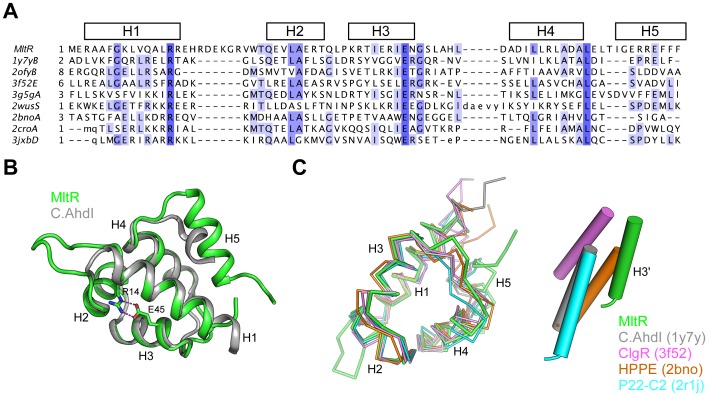
DNA-binding domain. (A) Structure-based, multiple sequence alignment between MltR-DBD and Xre family homologs. The secondary structural elements of MltR-DBD are shown at the top. Conserved residues are highlighted in blue. (B) Structural comparison between MltR-DBD (green) and C.AhdI (gray, PDB ID 1y7y). (C) Structural comparison between the MltR-DBD dimer (green) with other dimers from the Xre family: C.AhdI (gray), ClgR (PDB ID 3f52, pink), hydroxypropylphosphonic acid epoxidase HPPE (PDB ID 2bno, orange), and P22 c2 repressor (PDB ID 2r1j, cyan). The dimers were superimposed onto each other using one monomer of MltR-DBD as a reference. For clarity, only the recognition helices for the second monomer of the dimers are shown in a schematic tube-form.

Structural comparisons between MltR and homologs show that the MltR DBD is conserved (Fig. 4), particularly the first four helices (H1–H4). The DBD of MltR is most similar to the restriction-modification controller protein C.AhdI from *Aeromonas hydrophila* [17] (Fig. 4B, PDB ID 1y7y, DALI [18] Z = 10.7, rmsd 1.6 Å for 65 C_α_, sequence identity 31%). MltR contains a highly conserved, buried Arg-Glu pair (Arg14 and Glu45), which form hydrogen bonds and likely contribute to structural stability (Fig. 4A–B). The H1–H2 loop of MltR, which adopts a β-hairpin like structure, is longer than in the other related structures and displays the largest structural differences between different monomers in the asu, indicating that it is more flexible.

The dimeric arrangement of the DBD of MltR is consistent with dimers acting as the functional unit in the Xre family. The overall arrangement of the MltR dimer is similar to the dimers of other Xre family members, but the relative orientation of the H3 recognition helix (and, hence, the HTH motif) displays significant structural variation, as in other Xre family proteins [17], [19], [20], [21] (Fig. 4C). The difference in dimer conformation is mainly due to the fifth helix (H5), which mediates dimerization and displays more structural variability.

The distance between the recognition helices (H3) in the MltR dimer is approximately 34 Å, enabling the two recognition helices to fit into successive DNA major grooves by spanning ∼10 bp (1 turn of B-form DNA), and recognizing a two-fold symmetric DNA target (palindrome or pseudo-palindrome). The surface near the HTH motif is positively charged (Fig. 2B), providing additional evidence for its putative role in DNA binding. Thus, we conclude the general mode of DNA recognition by MltR is most likely conserved with other Xre family members.

### The Ligand-binding Domain in Complex with Myristic Acid

The C-terminal domain of MltR (Fig. 1) consists of an α/β profilin-like fold core with six strands that form an anti-parallel β-sheet (S1–S6, 621543 topology), and nine α-helices (H6–H14). Sequence analysis suggested that this domain might be remotely related to the PAS domain, which functions in many signaling proteins as a versatile signal sensor [22]. Indeed, the LBD contains a core similar to the prototypical PAS domain, which has a 21543 β-sheet topology (S1–S5), a helix before the first strand (H6), and a multiple-helix insertion between the second and third strands (H7–H13). Compared to a canonical PAS domain, the LBD contains a two-helix insertion (H12 and H13) between H11 and S3, as well as an additional helix-strand motif (H14–S6) at the C-terminus (Fig. 1A). From a structural similarity search with DALI, the LBD is most closely related to the PAS domain from a sensory box histidine kinase regulator from *Geobacter sulfurreducens* (PDB ID 3lug, Z = 7.7, Midwest Center for Structural Genomics, unpublished) with an rmsd of 3.2 Å for 104 aligned C_α_ atoms, despite very low sequence identity (6%). The LBD is also similar to the heme PAS sensor protein FixL (PDB ID 1ew0, Z = 6.4, rmsd 3.5 Å for 100 equivalent C_α_ atoms, seq id 10%) [23], and the photoactive yellow protein PYP [24] (PDB 3pyp, Z = 5.8, rmsd 3.4 Å for 99 equivalent C_α_ atoms, seq id 12%). The most conserved secondary structural elements among these proteins correspond to S1, S2, S4, S5, H6, H7, and H11 of MltR. Besides two common helices H7 and H11, the rest of the S2–S3 insertion in MltR differs substantially from other PAS domains. Interestingly, two helices within this region (H12 and H13) are involved in the domain interface between LBD and DBD, rather than in ligand binding. This structural evidence suggests that the LBD evolved from a PAS-like ancestral protein with adaption for binding to a different small-molecule ligand and acquisition of an interface with the N-terminal domain.

**Figure 5:**
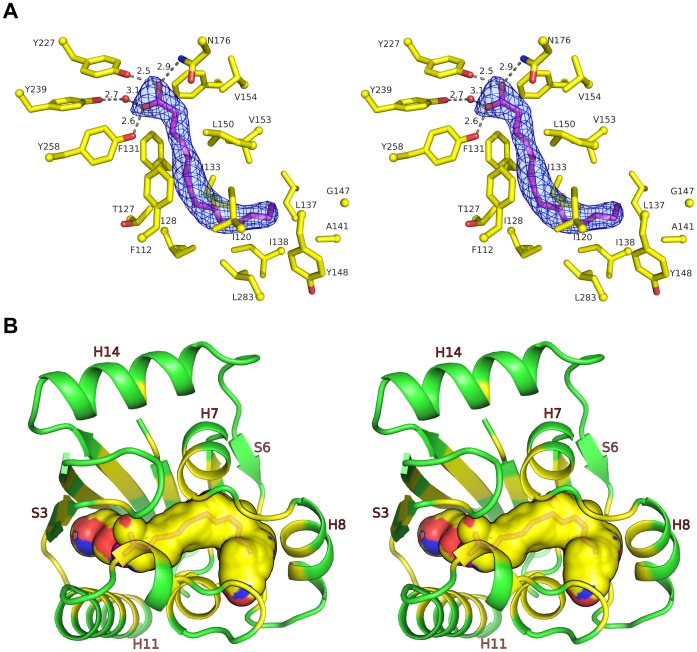
Ligand-binding site and ligand-protein interaction. (A) Interaction between myristic acid (MYR, magenta) and MltR (yellow). Hydrogen bonds are shown in dashed lines. The omit density map (Fo-Fc) is contoured at 3σ with an MYR modeled into the density. (B) Ligand binding cavity (shown as surface). Sections of the protein ribbon corresponding to residues contributing to the formation of the substrate or ligand binding cavity are colored yellow. The color of molecular surface of the cavity corresponds to underlying protein atoms (N: blue, O: red, C: yellow).

The small-molecule binding site of LBD is located between the β-sheet and the helical insertion between S2 and S3, which is also the common site for ligand binding in canonical PAS domains. Although MltR was crystallized without the addition of any substrate, additional electron density in the putative ligand-binding site clearly indicated the presence of a fatty acid-type ligand, which was modeled as myristic acid (MYR, Fig. 5A) based on clear, interpretable electron density. However, the exact nature of the natural ligand remains unknown. The electron density could also represent a related fatty acid or a mixture of fatty acids of different lengths. We cannot completely rule out binding of fatty acid-like impurities, such as penta-ethylene, glycol that may be present in the PEG 8000 that was used in the crystallization solution, but the fit of these to the electron density was not as good. The ligand sits in a location that has more similarity to the ligand binding site of photoactive yellow protein than to FixL. MYR interacts favorably with the protein such that its carboxyl group is stabilized by hydrogen bonding interactions with Tyr227, Tyr258, and Asn176, and its hydrophobic tail is surrounded by hydrophobic side chains in the interior of the protein. The average B-value of MYR (43 Å^2^) is similar to that of the protein (38 Å^2^). To our knowledge, this is the first report of a PAS-like domain or a prokaryotic transcription regulator that can potentially bind a fatty acid. Since the physiological role of MltR is currently unknown, the biological significance of the bound MYR is unclear. It is interesting to note that both MYR and the inducer for MmyB (2-alkyl-4-hydroxymethylfuran-3-carboxylic acids) are elongated molecules with hydrophilic heads and hydrophobic tails, suggesting that MYR mimics the physiological signal molecule.

Interestingly, the internal cavity of the LBD is formed by the β-sheet (S1–S6) and neighboring helices (H7–H11, and H14, Fig. 5B). The cavity spans the entire width of the LBD. MYR occupies most of the cavity with the head group at the center and the tail winding towards the surface. The cavity is not accessible from the protein surface by molecules larger than water. Thus, conformational changes are required for MYR (or other ligands) to gain access to the binding site.

### Structural Comparisons

**Figure 6:**
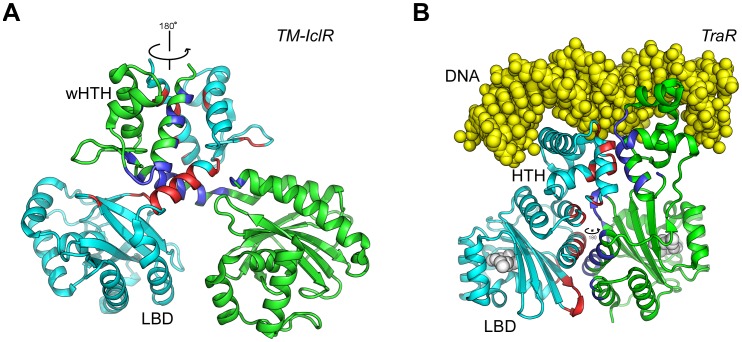
Structural comparisons between MltR, TM-IclR (PDB ID 1jmr), and TraR (PDB ID 1l3l). Dimers of (A) TM-IclR (unliganded) and (B) TraR (complexed with DNA, yellow vDW spheres), as illustrations of two proteins with similar domain compositions (HTH and PAS) to MltR. These dimers are shown in similar orientations as MltR in Fig. 2A (side-view). The dimer interfaces are also highlighted similarly as in Fig. 2A.

HTH and PAS domains are very common modules; however, database searches using the full-length structure of MltR did not identify any other proteins with a similar overall structure. A few known structures of transcription factors have a similar combination of HTH and PAS-like domains. For example, an IclR-type transcription factor from *Thermotoga maritima*, TM-IclR (TM0065), contains an N-terminal winged-helix-turn-helix (wHTH) domain and a C-terminal LBD with a profilin-like fold (PDB ID 1mkm) [25]. The LBDs of MltR and TM-IclR can be superposed with an rmsd of 3.8 Å for 103 C_α_ atoms, while the DBDs can be superimposed with an rmsd of 2.6 Å for 39 C_α_ atoms. Thus, the domain composition of IclR and MltR could be considered similar. Additionally, TraR from *Agrobacterium tumefaciens*, a LuxR family transcription factor, contains an N-terminal LBD and a C-terminal HTH domain (PDB ID 1l3l) [26]. The LBD of TraR is structurally similar to the LBD of TM-IclR, and both contain either an N-terminal or a C-terminal helix that packs against the “open” surface of the β-sheet of the prototypical PAS-fold. Also, in both TM-IclR and TraR, a dimer is required for DNA-binding. MltR differs from TM-IclR and TraR both in terms of the relative arrangement and interactions of the domains, and the mode of dimer assembly (cf. Fig. 2A and Fig. 6A–B).

The wHTH domain and the LBD of TM-IclR are connected by a helix and do not directly interact. The two monomers form a sort of “domain”-swapped or crossover dimer that only interact via the interface formed by the wHTH domains and the helical linkers. The two-fold symmetry of the wHTH domains does not extend to LBD; thus, the TM-IclR dimer in the crystal is asymmetric (Fig. 6A). As mentioned above, the HTH-containing DBD of TraR belongs to the LuxR family, which is characterized by a longer recognition helix, and shares only three equivalent helices with MltR (H1, H2, and H3). The DBD and LBD of TraR form a stable monomer with a more extensive domain interface compared to TM-IclR. TraR also forms a side-to-side dimer like MltR. However, the overall TraR dimer is asymmetric and, although the two-fold axes defined by DBDs and LBDs are perpendicular to each other, they do not intersect. The dimer interface involves helical contacts for both domains of TraR (Fig. 6B).

PAS domains are known to promote dimerization and oligomerization of many proteins [22], which they can accomplish in mechanistically different ways [27]. The β-sheet is frequently involved in the dimerization interface. The PAS sensor domain of a heme-regulated phosphodiesterase from *E. coli* (DOSH) dimerizes through perpendicular packing of the β-sheet (PDB ID 1v9z) [28]. In contrast, the LOV1 domain of phototropin 1 from *Arabidopsis thaliana* forms an antiparallel sheet across the dimer interface [29]. The perpendicular packing of the β-sheets in the MltR dimer is similar to DOSH. However, the orientation of the second sheet is related by an ∼180 degree rotation. Therefore, we conclude that MltR is a novel transcriptional regulator, based on the unique overall structure and the recognition of a fatty acid ligand.

### MmyB Family Regulators Occur Predominantly in Actinomycetes

The closest full-length homolog to MltR is from *Ktedonobacter racemifer* DSM 44963 (Krac_4648), which shares 26% amino-acid identity. We searched the sequence databases for additional homologs using profile-based method, which identified more than 1000 unique sequences with full-length homology in the UniRef100 dataset.

All of the full-length homologs of MltR identified were from bacteria. As indicated above, one of the best-studied examples of this emerging family is MmyB (∼20% seq id to MltR), the pathway-specific transcriptional activator for methylenomycin biosynthesis in *S. coelicolor* [10]. A few other (partially) characterized family members (Fig. S1) are related to antibiotic production. LlpRV is located in a gene cluster responsible for the production of the aromatic polyketide antibiotic lysolipin in *Streptomyces tendae* Tu 4042 [30]. CltP is present in the biosynthetic gene cluster encoding the thiopeptide antibiotic cyclothiazomycin in *S. hygroscopicus* 10–22 [31]. Additionally, Orf13 likely plays a regulatory role in the novel pathway of salicylate degradation by *Streptomyces sp*. strain WA46 [32].

Most of the family members are distributed in the actinobacteria (64%) and proteobacteria (29%) phyla with a few in firmicutes (3%) and chloroflexi (2%). Almost all MltR homologs from actinobacteria are from actinomycetes, except for three sequences from *Conexibacter woesei* DSM 14684. Most actinomycetes contain multiple paralogs. For example, 36 paralogs can be identified in *Streptomyces hygroscopicus* ATCC 53653, 21 in *Catenulispora acidiphila* DSM 44928, 16 in *Streptomyces coelicolor* A3 (2), 5 in *Streptomyces griseus* subsp. griseus NBRC 13350, and 10 in *Frankia alni* ACN14a. The *S. coelicolor* genome also encodes a protein (SCO6676) that consists of only the C-terminal PAS-like domain. However, MltR homologs are not universally present in actinomycetes, as illustrated by their absence in *Thermobifida fusca*. The number of orthologs found in genomes of proteobacteria is much smaller (one to six) with no identifiable homologs in model organisms, such as *E. coli* and *Bacillus subtilis*.

**Figure 7:**
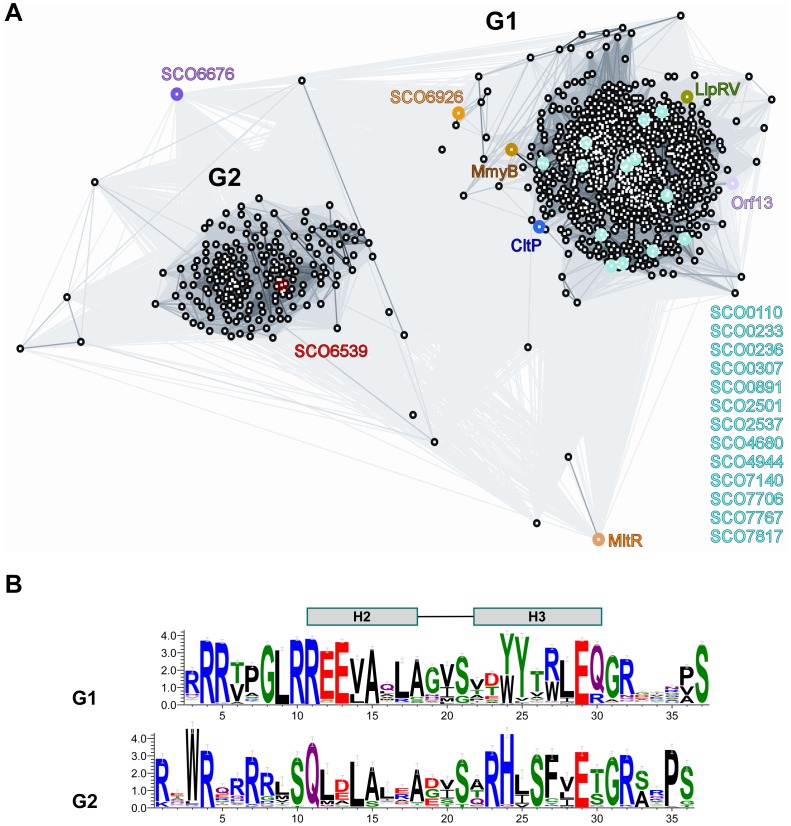
MmyB-like proteins define a new transcription factor family. (A) Two-dimensional projection of the CLANS [50] clustering analysis of full-length MmyB-like proteins. Each protein is indicated by a black dot. Lines indicate sequence similarity detectable with BLAST, and are colored by a gradient of grey according to the BLAST P-value ranging from the most significant (black, BLAST P-value ∼ 10^−200^) to the least significant (light grey, BLAST P-value ∼ 10^−4^). Characterized and additional family members from *S. coelicolor* are labeled and highlighted in different colors. (B) Sequence motifs of the HTH regions of the two groups (G1 and G2) within the family represented by sequence logo where the most frequently found residues are indicated at each position [52].

The clustering analysis of full-length sequences of MmyB-like protein homologs is shown in Fig. 7A. In general, the sequences can be divided into two clusters, one large group (G1, >800 sequences) and a smaller group (G2, ∼200 sequences). The full-length MltR is more closely related to G1, while the DBD region of MltR is more similar to G2 (see Material and Methods). 15 of the 16 paralogs in *S. coelicolor* are assigned to G1, while only SCO6539 belongs to G2. The proteins in G2 are more closely related to each other. Both groups contain a more conserved DNA-binding domain with a highly similar HTH motif (Fig. 7B) and a less conserved C-terminal PAS-like domain. The HTH motifs from each group share some common features, for example, the loop preceding H2 contains several highly conserved arginines (Fig. 7B). However, the distribution and nature of their conserved residues are distinct. The more divergent C-terminal PAS-like domains could be a result of adaptation for binding different small molecule inducers. Highly conserved residues in the LBD (e.g. Pro110, Ala111, Asn124 and Asn149) are likely structurally important. Furthermore, the domain interface contains several highly conserved residues (Glu70, Arg71 Arg181, and Trp210). These analyses suggest that the MmyB family may have evolved from a common ancestral protein. The proteins in subgroup G2 have less sequence variability indicating that they were likely evolved more recently or are functionally more critical to the survival of the organism. The more significant adaptations in their DNA- and ligand-binding regions likely affect the recognition of both DNA targets and “signals”.

### Functional Inference of the MmyB Family Regulators

To explore the functional diversity of the orthologs within a single species, we aligned the 16 paralogs occurring in the model organism *S. coelicolor* (Fig. S1) and analyzed their genetic environment. Interestingly, all regulators fall into either one of two classes, namely those that are immediately adjacent to a gene related to antibiotic production (eight, including *mmyB*) and those that are adjacent to a gene for an NAD(P)H dependent short- or medium-chain dehydrogenases/reductase (SDR or MDR) (Table S1). These enzymes form a highly diverse family, with alcohol dehydrogenase as the first studied example [33]. The suggestive linkage to SDR/MDR and antibiotic-related genes is also found in other actinomycetes; for example, the eight paralogs in the erythromycin producer *Saccharopolyspora erythraea* all share an upstream region with an oppositely transcribed gene, six of which encode an SDR, one a β-lactamase gene and one a hypothetical protein. Interestingly, the gene that encodes MltR, Caur_2278, is proximal to a gene coding an MDR (Caur_2281) and a β-lactamase domain protein (Caur_2280). Thus, the genetic association with SDR/MDR/antbiotics appears to be widespread. Therefore *mmyB* orthologs may form a very useful tool as genetic beacons for the identification of antibiotic-related genes in actinomycete genomes, analogous to the *mbtH* orthologs used as makers for the identification of gene clusters for specific types of nonribosomal peptide synthetases [2].

In 15 out of 16 paralogs discussed above (the exception being SCO7140), the MmyB-type regulatory gene shares its upstream region with, and is oppositely transcribed from, its putative target gene, with often only a small intergenic region separating them (Table S1). SCO0307 and SCO6926 are located next to a likely developmentally controlled gene, given that they contain a rare TTA codon [11]. SCO6925 and SCO6926 are immediately adjacent to a putative lantibiotic biosynthetic gene cluster and are flanked by very large noncoding sequences. Besides SCO6539, which belongs to the G2 cluster (see above), only SCO4944 is widely conserved in streptomycetes and several other actinomycetes. We therefore regard SCO4944 as the main member of the family in actinomycetes. An alignment of actinomycete orthologs of SCO4944 is shown in Fig. S2. Gene synteny analysis predicts that SCO4944 functionally relates to the adjacent SCO4945, which encodes a homolog of a mycothiol-dependent formaldehyde dehydrogenase in several streptomycetes. We anticipate that SCO4944 acts by regulating the transcription of SCO4945. Interestingly, in *S. griseus*, the orthologs (SGR_6891 for SCO4944 and SGR_6892 for SCO4945) are separated from *afsA* (*i.e.* SGR_6889), which is essential for the synthesis of the γ-butyrolactone A-factor (2-isocapryloyl-3R-hydroxymethyl-gamma-butyrolactone), by a single gene (SGR_6890). A-factor is a hormone-like signaling molecule that is required for streptomycin production, streptomycin resistance, and spore formation [34]. Microarray data show that the transcription of both SGR_6891 and SGR_6892 is induced immediately after addition of A-factor to liquid-grown cultures (within 5 min) [35], suggesting that both genes are part of A-factor regulatory cascade. However, lack of an obvious binding site makes direct repression by ArpA (the A-factor receptor protein) unlikely [35]. Since MmyB inducers are also A-factor-like signaling molecules, it is tempting to speculate that orthologs of SCO4944 and SGR_6891 might recognize A-factor or a similar effector molecule. Overall, both experimental results and bioinformatics analysis suggest that MmyB family regulators in actinomycetes may play important roles in secondary metabolite and fatty acid metabolism.

### A Model for Signal-activated DNA-binding

**Figure 8:**
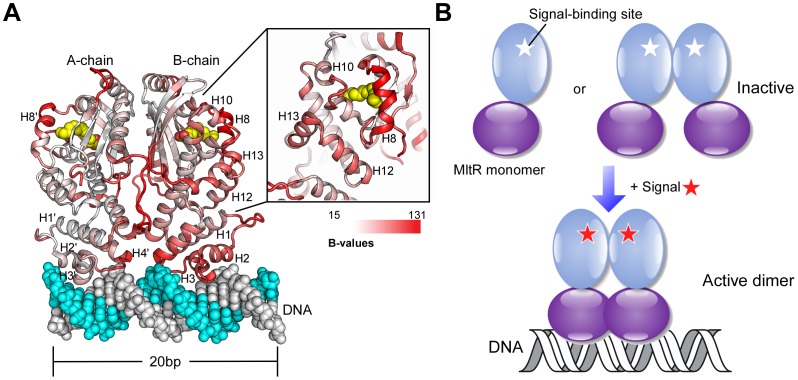
Model for ligand-induced DNA binding by MltR. (A) Model of MltR bound to DNA. MltR is colored as a gradient corresponding to B-values from low (white) to high (red). The fatty acid ligand molecules are shown as yellow spheres. (B) Schematic representation of proposed activation of MltR by ligand binding. Ligand (or signal, shown as a red star) binding may induce conformational changes in MltR to promote formation of a stable dimer, and reorient the DBD for DNA interaction.

Our analysis indicates that MltR most likely functions as a dimer when binding to a DNA target, which is supported by modeling. We docked the MltR dimer onto an 20-base pair fragment of ideal B-DNA based on shape complementarity using PATCHDOCK [36]. The resulting model (Fig. 8A), is similar to other Xre-type DNA-binding domain-DNA complexes, such as lambda repressor [37] and P22 c2 repressor [21], suggesting that it is possible for the observed MltR dimer to bind DNA without significant structural changes. In the model, two adjacent major grooves of the DNA are contacted by the two putative HTH reading heads. The H1–H2 loop and the N-terminus of H2 may also contribute to DNA binding as they are in close contact with the minor groove. Furthermore, the H3–H4 loop is located close to the backbone of DNA. The LBD does not make contacts with DNA directly. The MYR binding site is ∼50 Å from the DNA. This model supports the hypothesis that the DNA-binding activity of MltR is regulated by the LBD and is consistent with the mode of action of MmyB[7], [9] .

The PAS domain is a common module involved in regulating adaptive responses of the cell, achieved through its ability to transmit a signal from the receptor site to other domains or partners through conformational changes. Structural and computational simulations suggest that the PAS module is intrinsically dynamic [27]. The structural flexibility of the ligand-binding site plays a central role in promoting conformational changes, which is then propagated to other domains or partners through domain interfaces [27]. The ligand-binding site and the DBD of molecules B and C in the asu are not involved in the crystal packing. Their B-values are significantly higher than the central β-sheet (Fig. 8A), suggesting that these regions may be even more flexible in solution. Here, we propose a model of ligand-induced activation of MltR based on the dynamic nature of the PAS domain (Fig. 8B).

It is assumed that MltR can exist in two states: a ligand-free inactive form and a ligand-bound active form. In the inactive state, the ligand-free form would exist in an “open” conformation, which would undergo conformational changes upon ligand binding, resulting in the occluded, active ligand-bound form (“closed” conformation). To achieve this conformational isomerism, ligand-free MltR may sample “open” and “closed” states through conformational changes in helix H8 and surrounding regions (Fig. 8A). This movement in LBD may propagate to the DBD through helices H12 and H13. A dynamic DBD would in turn affect the dimer interface, and potentially the oligomeric state, such that MltR may exist as a monomer or a weak dimer in a ligand-free state (Fig. 8B).

Therefore, the ligand-free state likely impairs DNA-binding. We anticipate that upon binding of the physiological ligand to LBD, the molecular dynamics would shift towards the “closed” state, which would stabilize the conformation of the DBD, promote dimerization and allow MltR to bind the DNA target. In other words, ligand binding may contribute to the formation of a more stable dimer, and help orient the DBD domain into a productive DNA-binding conformation. This model of ligand-induced activation shares similarities to proposed mechanisms for the PAS sensor domains in signaling pathways, such as the heme-regulated phosphodiesterase [28].

In conclusion, we have determined the crystal structure of a novel transcription factor that is a representative of a large group of regulators with significant implications in antibiotic production and fatty-acid biosynthesis. These regulators appear to function as transcription activators that are switched on by the accumulation of small-molecule signals, such as precursors of the underlying biosynthetic pathways. The structure provides insights into the mechanisms of activation and DNA binding. These results lay a solid foundation for future characterization of this emerging new protein family, and may prove to be useful in the hunt for novel gene clusters for antibiotic production in actinomycetes.

## Materials and Methods

### Cloning and Protein Purification

Clones were generated using the Polymerase Incomplete Primer Extension (PIPE) cloning method [38]. The gene encoding MltR (GenBank: YP_001635876.1, UniProt: A9WGF5_CHLAA, locus name: Caur_2278) was amplified by polymerase chain reaction (PCR) from *C. aurantiacus J-10-fl* genomic DNA using *PfuTurbo* DNA polymerase (Stratagene) and I-PIPE (Insert) primers (forward primer, 5′-ctgtacttccagggcATGGAACGAGCAGCTTTTGGCAAACTGG-3′; reverse primer, 5′-aattaagtcgcgttaGCCATTACTGCGCGGCCATGGGGCGAG-3′, target sequence in upper case) that included sequences for the predicted 5' and 3' ends. The expression vector, pSpeedET, which encodes an amino-terminal tobacco etch virus (TEV) protease-cleavable expression and purification tag (MGSDKIHHHHHHENLYFQ/G), was PCR amplified with V-PIPE (Vector) primers (forward primer: 5′-taacgcgacttaattaactcgtttaaacggtctccagc-3′, reverse primer: 5′-gccctggaagtacaggttttcgtgatgatgatgatgatg-3′). V-PIPE and I-PIPE PCR products were mixed to anneal the amplified DNA fragments together. *E. coli* GeneHogs (Invitrogen) competent cells were transformed with the I-PIPE/V-PIPE mixture and dispensed on selective LB-agar plates. The cloning junctions were confirmed by DNA sequencing. Expression was performed in a selenomethionine(SeMet)-containing medium at 37°C. SeMet was incorporated via inhibition of methionine biosynthesis, which does not require a methionine auxotrophic strain. At the end of fermentation, lysozyme was added to the culture to a final concentration of 250 µg/ml, and the cells were harvested and frozen. After one freeze/thaw cycle, the cells were homogenized in lysis buffer [50 mM HEPES pH 8.0, 50 mM NaCl, 10 mM imidazole, and 1 mM Tris(2-carboxyethyl)phosphine-HCl (TCEP)] and passed through a Microfluidizer (Microfluidics). The lysate was clarified by centrifugation at 32,500×g for 30 minutes and loaded onto a nickel-chelating resin (GE Healthcare) pre-equilibrated with lysis buffer, the resin washed with wash buffer [50 mM HEPES pH 8.0, 300 mM NaCl, 40 mM imidazole, 10% (v/v) glycerol, and 1 mM TCEP], and the protein was eluted with elution buffer [20 mM HEPES pH 8.0, 300 mM imidazole, 10% (v/v) glycerol, and 1 mM TCEP]. The eluate was buffer exchanged with TEV buffer [20 mM HEPES, 200 mM NaCl, 40 mM imidazole, 1 mM TCEP, pH 8.0] using a PD-10 column (GE Healthcare), and incubated with 1 mg of TEV protease per 15 mg of eluted protein for 2 hrs at ambient temperature and then overnight at 4°C. The protease-treated eluate was passed over nickel-chelating resin (GE Healthcare) pre-equilibrated with crystallization buffer [20 mM HEPES pH 8.0, 200 mM NaCl, 40 mM imidazole, and 1 mM TCEP] to remove the His-tagged TEV and purification tag and the resin was washed with the same buffer. The flow-through and wash fractions were combined and concentrated to 11.1 mg/ml by centrifugal ultrafiltration (Millipore) for crystallization trials.

### Crystallization and Diffraction Screening

MltR was crystallized using the nanodroplet vapor diffusion method [39] with standard JCSG crystallization protocols [13]. Sitting drops composed of 200 nl protein solution mixed with 200 nl crystallization solution in a sitting drop format were equilibrated against a 50 µl reservoir at 277 K for 29 days prior to harvest. The crystallization reagent consisted of 0.2M NaCl, 10.5% polyethylene glycol 8000, 0.1M CHES pH 9.1. Ethylene glycol was added to a final concentration of 8% (v/v) as a cryoprotectant. Initial screening for diffraction was carried out using the Stanford Automated Mounting system (SAM) [40] at the Stanford Synchrotron Radiation Lightsource (SSRL, Menlo Park, CA).

### Analytical Size Exclusion Filtration Analysis

The oligomeric state of MltR in solution was determined using a 0.8×30 cm^2^ Shodex Protein KW-803 size exclusion column (Thomson Instruments) equilibrated in 20 mM Tris-HCl, 200 mM NaCl, 0.5 mM TCEP at pH 7.5 and pre-calibrated with gel filtration standards (Bio-Rad) [38]. The molecular weight was calculated using ASTRA 5.1.5 software (Wyatt Technology).

### Data Collection, Structure Solution, and Refinement

Single-wavelength anomalous diffraction (SAD) data were collected at wavelength corresponding to the peak of a selenium SAD experiment at 100 K using Mar CCD 325 detector (Rayonix) at SSRL beamline 11-1. The data were integrated and reduced using XDS and then scaled with the program XSCALE [41]. 32 selenium sites were located with SHELXD [42]. Phase refinement, density modification and automatic model building were performed using autoSHARP [43] (FOM 0.29) and Buccaneer [44]. This automated process produced an initial model that was 80% complete. Further model completion were performed manually with Coot [45] and refined with BUSTER [46]. TLS parameters were refined with each monomer as a rigid body group. Non-crystallographic restraints were applied throughout the refinement using BUSTER LSSR implementation (AUTONCS). Experimental phases in the form of Hendrickson-Lattman coefficients were used as restraints during refinement. Data and refinement statistics are summarized in Table 1. Analysis of the stereochemical quality of the model was accomplished using MolProbity [14]. All molecular graphics were prepared with PyMOL (http://www.pymol.org). The electrostatics were calculated using APBS [47]. The structure factors and atomic coordinates of MltR have been deposited in the Protein Data Bank (http://www.pdb.org/) with PDB code 3pxp.

### Bioinformatic Analysis

In order to identify homologs of MltR, we collected the top 57 hits with full-length similarity to MltR from a BLAST search against UniProt UniRef50 (UniProt Reference Clusters; http://www.ebi.ac.uk/uniref/) dataset (E-value cutoff 1.0e-2). A sequence profile was then built using the HMMBUILD program of HMMER (version 3.0) [48], based on a multiple sequence alignment produced by T_Coffee [49]. The generated profile is then searched against public sequence databases UniRef100 dataset using HMMSEARCH program of HMMER. The sequences were analyzed using the CLANS program [50], which clusters sets of protein sequences using the P-values of high-scoring segment pairs (HSPs) obtained from an all-to-all BLAST search using a version of the Fruchterman-Reingold graph layout algorithm. To estimate relationships between MltR and the resulting G1 and G2 clusters, profiles of these clusters were built using HHBUILD and queried using the full length and the DBD region of the MltR sequence (G1: E_MltR vs. G1_ = 3e-22, E_MltR-DBD vs G1_>10; G2: E_MltR vs G2_ = 4.2e-17, E_MltR-DBD vs G2_ = 1e-08) using HMMSEARCH. Multiple sequence aligments were generated using ClustalW [51]. The sequence logo was produced by WebLogo [52].

## Supporting Information

Figure S1 **Multiple sequence alignment of MltR and highlighted homologs from** **Fig. 7A** **.**
(PDF)

Figure S2 **Multiple sequence alignment of SCO4944 and orthologs from representative actinomycetes.**
(PDF)

Table S1 **Members of the MmyB family Xre-type regulators found in** ***S. coelicolor*** **A3(2).**
(PDF)
