# Correction: Structure of an MmyB-Like Regulator from *C. aurantiacus*, Member of a New Transcription Factor Family Linked to Antibiotic Metabolism in Actinomycetes

**DOI:** 10.1371/annotation/f345da14-66b8-4e8b-b448-576dc29a03f5

**Published:** 2012-08-02

**Authors:** Qingping Xu, Gilles P. van Wezel, Hsiu-Ju Chiu, Lukasz Jaroszewski, Heath E. Klock, Mark W. Knuth, Mitchell D. Miller, Scott A. Lesley, Adam Godzik, Marc-André Elsliger, Ashley M. Deacon, Ian A. Wilson

The following information was missing from the Funding section: This work was supported by the NIH, National Institute of General Medical Sciences, Protein Structure Initiative [U54 GM094586 and GM074898]. The funders had no role in study design, data collection and analysis, decision to publish, or preparation of the manuscript. 

