# Supplementary material for: Structure of an MmyB-Like Regulator from C. aurantiacus, Member of a New Transcription Factor Family Linked to Antibiotic Metabolism in Actinomycetes
Source: PLoS One. 2012 Jul 26;7(7):e41359. doi: 10.1371/journal.pone.0041359 (PMC3406030; doi:10.1371/journal.pone.0041359)
Supplement: Figure S1 — Multiple sequence alignment of MltR and highlighted homologs from Fig. 7A . (PDF) [file pone.0041359.s001.pdf]

```
MltR            -------------MERAAFGKLVQALRREH---------------RDEKGRVWTQEVLAER
SCO7140         ------------MADRAALAAFLRARREALQPEDVGLP---RG-RRRRTGGLRREEVAAL
SCO2501         (8)QSPPRDTGSEIRRHELAAFLRSRRERIAPEQVGLP---RG-RRRRTPGLRREEVAQL
SCO7817         ------------------MADFLRHRREALRPEDVGLS---PG-ARRRARGLRREEVAAL
SCO0307         --------------MSSELGDFLKARRRELSPPTAGLP---AD-GRRRVPGLRREEVAVL
SCO0110         ---------MPTNTNRAELRDFLRSRRARVSPEDVGIP---AT-VRRRTPGLRREEVAQL
SCO2537         --MITRAGTAGRDRRRSELREFLMSRRARVSPAEVGLP---DGGARRRTPGLRREEVAVL
SCO7767         -------------MSSQELADFLRRRREDLRPEDVQAE(5)SR-RARRTPGLRREEVAAL
SCO0236         ----------MKDESGNRLGSYLRARRELITPAQAGIP---PG-GNRRVPGLRREEVALL
SCO4680         -------------MKYAELGSFLRSRRERIRPADVGLP---AG-PRRRVPGLRREEVAHL
SCO7706         -------------MDDNHLGEFLRARRAGLRPQDVNMA---SH-GLRRVAGLRREEVAVL
SCO0233         ------------MIDRSGLAQFLRHRREALQPEDVGLP---RG-RRRRTSGLRREEVAAL
SCO0891         -------------MTATEFGRALRLRRDRVSPEAAGLP---AG-GHRRAAGLRREELALL
SCO4944         --------MDGQLDRRAELSEFLRTRRARLKPEDVGLE---SYGRQRRVPGLRREELAQL
MmyB            (8)KD-STVCSPKRQREALRHFLRSRRARLSPDDVGLL---AT-GRRHTPGLRREEVAVI
LlpRV           -------------MDDNHLGDFLRARRAALRPHDVGMP---SH-GTRRVAGLRREEVAVL
CltP            ------MIVAMADVRSRELGAYLRARRDRLSPADVGLP---DGGGRRRVKGLRREEVAVL
Orf13           ------------------------MCRAQLSPRTVGLP---ESRAPRRVPGLRREEVAQL
SCO6926         --MCGAIKDGGGEGESTGIGALLRVFRAAA--------------GTTLGRPILQREVADA
SCO6539         ---MTALVSGTSPATDRGVGPMLRAWR--------------------ERRRVSQLELALR
Consensus/80%   ......................LR.RR....P..............RR..GLRREEVA.L

MltR            TQLPKRTIERIENGSLAHL-DADILLRLADALELTIGERREFFFAATGIIEQKSA----T
SCO7140         CDMSVDYYSRLEQPRGPHP-SEQMLTSMARGLRLSLEERDLLFQLAGHALPRRA-----R
SCO2501         SAVGVTWYTWLEQARDIQV-SVQVLDALARTLLLDPTERAHLFQLAGSVDPTPA-----T
SCO7817         ALMSTDYYTRLEQRRGPQP-SERMLDSLARALRLTRAERDYLYRVAGHNAPASL-----S
SCO0307         ASISPGHYTRLEQGRR-RA-SEPVLDALARALRLSADERAYLFELSGKDAGRPLG----R
SCO0110         AGVGITWYTWLEQGRPINV-SSQVLDAVARTLILDAAERDHLYRLAEVPQPPVIS----D
SCO2537         AGVGASWYQWLEQGRDISV-SPQVLDAVGRVLRLSNTERRHLYVLAGLNPPAAEV--E-P
SCO7767         AQVSVSYYERLEQARAPRP-SPQVLSALATALQLTDAERDHLARLAGQVLPAEND----G
SCO0236         AGISPDYYLRLERGRDKNP-SPQVLESLARVLQLDDIERTYLLGLAAARPRAPRR----K
SCO4680         AGASVDYYNELERGAGSQP-SEQMLAALARALRLSADERDYLYRLADRPVPVPGG----P
SCO7706         AGVNADYYTRLEQGRERHP-SPQVLDALGRALRLDPEARAHLHRLAGVSPAGRDS--L-H
SCO0233         CHMSTDYYARLERERGPQP-SQQMIVSIAQGLHLSLDERDHLFRLAGHTPPPRG-----T
SCO0891         AGISVDYVTRLEQGRAANP-SAQVVEALVRALRLPAEERAHLFRLAGLAPPGPE-----A
SCO4944         AGVSVAYYTRLEQGNGQNV-SAEVLDAIARALRLTDAEQAHLTHLARPKQQRRRAGAA-R
MmyB            AGVSASWYTWLEQGRDIKV-SDGVLNAISQALRLDDTERAHLYRLAGVNPPQSVP--A-T
LlpRV           AGVNADYYTRLEQGRERNP-SPQVIDALGHALRLDEDARTHLYRLAGATPGERPS--P-H
CltP            ANVGSSWYTWLEQGRDVRP-SVEVLAAIADALRLSSSERRHLFLLGGHPDAEP-P----S
Orf13           AGISTDRYTRWEQGRA-PV-PTSVLATLVRVLHLDDDQRDHLFALAAKVECAPRR----R
SCO6926         LHRSERWYRDLEGGVITRPLTRHELDTIGTLLGLDRVQRRALFLVSNGGGLSSPE--T-Q
SCO6539         ADSSARHISFVETGRS-RP-SEEMVLRLAEHLDVPVRERNALLLAAGYAPHYPETPLDDP
Consensus/80%   .......Y..LE..R.....S...L......L.L...ER..L..LA..............

MltR            YKRSPEESLQYLIDMIRNMNVPAFVTDQYVNIIAANMITIRFFNIPMEL(6)LPHGYNLM
SCO7140         RGDHVAPGTMRILDRL--EDTPAQVMNHLGETLSQTRPAMTLLGDQTAY---TGLARSSH
SCO2501         DCPAITPAVRALLEQF--EPYPACVQNSRYDILAHNRTYGLLLCDLDAV---PPEDRNCM
SCO7817         DPSPVAPALLRVLDRL--DDTPALILTELGETLVQNRMAVALFGDASRH---TGLARSAV
SCO0307         PGRQVRPQLRRLLDDL--THTPALVLGRCTDILAWNPPAAALFTDFALL---SRDERNFV
SCO0110         PPTELPGHLDTVLEAL--DPLPAMLVDARTDVLRWNRAYAALHPALVSA---PPGKRNTL
SCO2537         SKRDMCEGLRRLIDTW--MPYPAHIMDRYYNCVLYNDAAATVLGM---R---PETTWNCI
SCO7767         TPEHVPEDAQQLLGRL--DGIPAYIVNDRQDIVAWNAAAAALITDFSRL---TPDERNLT
SCO0236         RPEHVPARVHELLA-H--LPIPAFVEGRAFDVLASNPMAVALSPRLR-------PGQNRL
SCO4680         -ASHVHPGMLDLLGRM--TSTPAQVITDLHVTLVQNPLAVALLGDQSGY---RGPRASFV
SCO7706         ATERVGPALRQLMDGY--AHTPAFVMSRTLDVLAANALADALYAPFT-------PADNLA
SCO0233         DGEHISPGLLRILDRL--DDTPAEIVTELGETLRQTRLGIALTGDQTRY---SGPARSIG
SCO0891         VPAYIPPSVHRLLDRL--AGTPVAVYDAMWTLLLANPPYAALMGDPSEW---HGPERNGV
```

```
SCO4944          GKQPVRVALLQLLDSI--DTVPAYVSGRRSEILAWNRMAAALFGDWGKL---PAAERNWA
MmyB             AGQTETSRLQLIVDGW--LPAPAFVVDRYWNTLAANQAARSALGV-------GAGDQNYL
LlpRV            SAGRVSPALRQLMDGY--PHTPAFVMNRTLDLLAVNALAQALYSAFT-------PADNLA
CltP             NCEGTSGRVQTLLDSL--SPHPAVVMNPWFEPLAYNAPFRFMIDDLEAL---PPADRNCA
Orf13            PAQKVQPQLGRLLDEL--TGTPALVLGRHMDILAWNALAAALLTDFEHI---PEKKRNYA
SCO6926          EPPRISDELRLLLDQ---QPFPAYVIDATWNVLAVNTSMAALFPWS------TAPGANLM
SCO6539          ALGALREGMERLIRGY--EPYPALVVDATYRVLAANRGIAMLMDGVAEH--LLTPPLNAI
Consensus/80%    .....................PA.........L..N.....L...............N..


MltR             RVVFGTEYDFRRVVGT--MWDEVARHNMQLFRAISLRV-RADGYFVELLDNLM-QYREFK
SCO7140          YRWFTDPAA--RLVHPASDHAEQSRLMVADLHSAYSRD-GGDSGAAALVDALNRESPEFA
SCO2501          VLCFTHEDW--RSSIV--HLEETQRLMAARFRATMAGH-LAEPAWKMLLKRLRTQSPAFR
SCO7817          YRWFTDPSE--RSVYPESDWGRQSRAQVANLRAAYGMR-GARSHAGALVRILQQSNEEFA
SCO0307          RLVFRDPAV--RALYT--DWPYMARACVAQLRMEAARD-PHDPRLSALVAELSAQDPDFR
SCO0110          WQLFAAPEG--QPRIV--NRDEQAPEAVAGFRYRYSQN-AGDPRWQDFVARLRTASPLFS
SCO2537          LDFFTDPLY--RSRAR--NWEHNARTVVAQFRATCAAN-PDDEGFQQVLADLTGASAEFA
SCO7767          RISTRFRGTLCTGAPG--SESEFSQQVAAQLRAASVLY-PTDKVLAELINEFATHDPDFA
SCO0236          RDLLLDPEE--QEFHQ--DWPKATADFVAALRTTIGDD-TDNPRFVELVGELSLSSQRFR
SCO4680          HRWFTEPEA--RLLYPEADHAYQSRSFVADLRAAAARRDAKDTEAGAMVRTLLGVSPEFA
SCO7706          RMIFLDPAG--REFYQ--EWDRAAQAVVADLRQAHGFD-PEHPRLRRLVDTLTEHSAVFR
SCO0233          YRWFTDPAT--RQLYHPDDHAFLTRMFASGLREVATLR-GPGSRAAHYADLLLAQSEEFR
SCO0891          WRNFVGPGS--RARYTPGERRSFESALVADLRATAARY-PADRRLGRLVAELRARSERFA
SCO4944          RLVFLNPDY--RELFV--DWDQKAYDMVSFLRMDAGRH-PDDPRLSALVGELSVKSEEFR
MmyB             AAFFTEPTA--RARYL--DWDKLATRLVGQFRVQAARF-PEDPRFDRIARQLCATDHAFA
LlpRV            RMIFLDPAG--PAFYT--DWDRAAQAVVANLREATGFA-PDDPRLRELVGTLTEHSPDFA
CltP             YLHFTHPDW--IAAHS--DHEQECAAVVAKLRAYHGES-VTDPAWAPLLARLREESPLFV
Orf13            RLLFTDPGF--RVLYP--DWRTIARSCVAHLRVEAAQC-PGDPELTALVGELSVADADFR
SCO6926          RWLLLSAEA--RDQHL--HWEADAEVCVCMLRDAAVDR-PHDPDLQQLISDSR-QNPAVR
SCO6539          RLTLHPDGL--APRIR--NLREWRGHLLEQMERQIALH-R-SRPLRELYDEVAAYPVPES
Consensus/80%    ...F.........................R...................L......F.


MltR             RFWERAHLETEDTSA(4)YQYTHPVYGLLSYVSSRSQIPT-S---MGLLSMHTYIPLSPA
SCO7140          GLWRQRPVLGPYCAS---KRFVHPQVGTLELHCQTLIDPD-H---GQRLVVYTATPGTES
SCO2501          EAWERHEVVAHRGKR---KEFLNRHVGRIRVDHTDLWLGPEP---GPRMVTYAPADED-S
SCO7817          ELWERHEVAQRFEDH---KTLIHPEVGAIEVDCQALFTED-Q---SQTLLVLTAPPRTES
SCO0307          TWWKDHRVAVRGAGT---KDLRHPVIGELTLDWSALTDTADP---DQQLIALTAAPGTPS
SCO0110          RLWTTHDVAPPHLCD---KRYDITGIGEVSLRATSMELTDHP---GVRLVVQTPADRR-S
SCO2537          ALWEERDIEDAGQIR---KELDHPLVGLLSLESTALQVPARP---DLTIVLHTPLEEANT
SCO7767          SSWRNHAVRPIPGVR---KRLHHPTLGELEIDRHTLSLPG-S---GFSLVMYTAEVGSPS
SCO0236          TLWARHEVRSLDGGS---TTVHHPVVGDLHLHRDKLPVE------DVILVVYYPDKDSDS
SCO4680          ALWADHDVAFRRHDR---KRLVHPVIGLVEVNCLNLFSED-G---RQRLLWFTPAVGTES
SCO7706          RLWAEHSVRGKTQDA---KRLLHPDVGTLSLTYQSFDVRDAP---GQQLVIYHAEPGSPS
SCO0233          RVWKEHTVGIRPKEV---KHFVHPEVGALELTCQTLLDPS-Q---AHMLLVYTATPGGES
SCO0891          ELWRADAVGRHEAAR---KTIDHPRVGPVVLDCDVLGVAG-S---DLRIMVYTAEPDTAD
SCO4944          RLWATHDVKEKSYGV---KRMRHPLVGDLTLSFETFRLVDDD---EQAFITYHAEPGSPS
MmyB             DLWARHETCDTAMTS---VRVRPPGEESMRFEHLILALLENA---DLRLMLYMPRGAHIS
LlpRV            RLWRSHTVRGKTQDA---KRFLHPDIGPLTLTYQAFDVRETP---GRQLVIYHAEPGSPS
CltP             RLWEQADVSTEPGRV---KRIRSRHVGSLSVRAITLLLEENP---RTRVVVYQPADR-TT
Orf13            QWWAGRQVPYLQVGS---KRLSHPVAGDLTLDWDSLTSTADP---AQQLVIMTPEPGTPS
SCO6926          DLWTRGAADFADHYD---GHVLQ---------MTLPLFDGQ---VTELVTHVLQPAGLP
SCO6539          VPGAEPDEPVPYFAL---PMQIEHEGHVLSFISSISTFNTPMDVTVAELAIETLLPADPA
Consensus/80%    ..W.......................G.................................


MltR             TTDLFAKLSTVANQDVIRLAPWPRSNG---------
SCO7140          HTNLRLLSLLPVS-----------------------
SCO2501          RERLERLHAIALEREPAASG----------------
SCO7817          HEKLQLLAVLGLHRFTEAEDQVR-------------
SCO0307          HDGLRALRDLGAVTSGRTGPGVPGGGGEIAGASAQ-
SCO0110          RENIDRLLRQATHL----------------------
SCO2537          AAKLEWLASPEGRRGAMYPVAG--------------
```

```
SCO7767          AAALKSL-----------------------------
SCO0236          DEKLRLLAGLSHSESAGTPRSGSSTGGRPPQEE---
SCO4680          AGLLELLSVVGTQEVSEVSAATRTAGSSTRRQG---
SCO7706          AHALGLLGSLHAGERRQGASDSAGN-----------
SCO0233          YEKLQLLSVIGAQTLR--------------------
SCO0891          AERLALLTVLGTQELVE-------------------
SCO4944          ADALRLLASWGTDAAASLPA----------------
MmyB             AEAGLRLVPADPLTRNSLLQLPGRPMTSRPVER(4)
LlpRV            AQSLNLLGSLHATRSQADSPHVAGHPIRRGDRHR--
CltP             QERLEELAHRIARGTIDGPARVRHLLAAT-------
Orf13            HDGLRFLASWTAAPYERARDTTA-------------
SCO6926          GCRMTILTQRAPQEPSGRQPVQAT------------
SCO6539          TVKYLQTLLP--------------------------
Consensus/80%    ...L..L.............................
```
