# Supplementary material for: Structure of an MmyB-Like Regulator from C. aurantiacus, Member of a New Transcription Factor Family Linked to Antibiotic Metabolism in Actinomycetes
Source: PLoS One. 2012 Jul 26;7(7):e41359. doi: 10.1371/journal.pone.0041359 (PMC3406030; doi:10.1371/journal.pone.0041359)
Supplement: Figure S2 — Multiple sequence alignment of SCO4944 and orthologs from representative actinomycetes. (PDF) [file pone.0041359.s002.pdf]

```
SCO4944         --MDGQLDRRAELSEFLRTRRARLKPEDVGLESYGRQRRVPGLRREELAQLAGVSVAYYT
SACE_1844       ------MDHRTELSQFLRTRRARLSPADVGLPRYGR-RRVPGLRREELAQLAGVSVAYYT
FRAAL2385       (6)AGNTGRMTELGDFLRSRRAQLTPEDVGLSRYGERRRVPGLRREEIAQLAGVSVTYYT
KSE_00670t      ------MDTPSVLGDFLRSRRARLQPEDVGLRAYGARRRVPGLRREELAQLAGVSVTHYT
caci_7725       (8)DRRIDRRAELSEFLRSRRARLNPGDVGLPDYGGRRRVPGLRREELALTAGVSVDHYV
AMED_8063       -----MMSDAAELGAFLKARRAALDPADLGLPPGVTQRRVKGLRREELAQLAGISVDYYT
Micau_3909      -----MAG-HSDLGRFLRQCRADLTPEAVGIAAVAQGRRVRGLRREEVALLAGVSVDYYT
Amir_0367       -----MAQNNAELADFLKRARAQGDPERAGLPPDGRVRRVPGLRREEVARLAGVSTDYYT
Msmeg_0523      -----MSDRKTELAEFLSARRARLTPEEVGLEQPGTRRRVPGLRREELALLAGVSVDYYT
Sros_3720       ------MDHNAELREFLRTRRARLSPQQAGVPATGGTRRVPGLRREELARLAGVSVDYYT
Aaur_2039       ------MDNRTETRDFLATRRAKITPEQAGLAAYGGNRRVPGLRRGEVAMLAGVSVEYYT
Krad_2718       ---MAVMDNRAEVRDFLTTRRAKLTPAEAGLPDVG-PRRVPGLRRAEAAALAGISVEYYA
Gobs_1568       ---MVAVDTRKDLRDFLATRRARITPQQAGLPTYGGHRRVPGLRREEVALLAGVSVEYYT
RO00570         ---MSSTDLRTEIREFLSTRRARITPEQSGLPVFGGNRRVKGLRREEVAMLAGVSVDYYV
CgR_0165        ------MNLHSDAREFLKSRRDRVTPQAAGLPSYGRNRRVPGLRREEVALLAGVSVDYYT
Consensus/80%   ...............FL..RRA...P...GL...G..RRVPGLRREE.A.LAGVSV.YYT

SCO4944         RLEQGNGQNVSAEVLDAIARALRLTDAEQAHLTHLARPKQQ----RRRAGAARGKQPVRV
SACE_1844       RLEQGHAQNVSGEVLGAICDALRLDQAERDHLRNLVRPVRK----RRRS----AQQRVRP
FRAAL2385       RLEQGQSRNASDAVLDALARVLRLTDEETAYLHGLTRPAAA----RRAS----RPEHVRP
KSE_00670t      RLEQGQSTNASDAVLDAIARALRLNTDETAHLRDLARPAAP----ARPAPL--RPDYARP
caci_7725       RLEQGRAVQFSEEVLDAVARALRLDPTECEHLYRLARPYSAQVY-QGTGKR--EPQKVRP
AMED_8063       RLEQGRAKNVSEAILGALARALRLDAGEESYLRNLAAP-------KRREKS--APQRVRP
Micau_3909      RLEQGRHTSPSAAVVDALARVFRLDAAARAHLADLARPAR-----RSSRQ--MPAQRVRP
Amir_0367       RLEQGRRIVPSPAVVDALGRALGLDDAGQAHLRDLIGVGG-----PVGRRRTTGAQRLRP
Msmeg_0523      RLEQGRSRSASADVLDALAAALQLDDAERAHLHTLAKPQ------PAQRKRRSKPQMVDP
Sros_3720       RLEQGRHLNVSESVLDAVARALRLDGVERAYLFELSRSKPR----PTRRRAPTRPQRVRP
Aaur_2039       RLERGNLSGVSESVLESLAGALQLDDAERAHLYDLARAASDG---GKSRRRPVRKQAIRP
Krad_2718       KLERGALAGVSASVLESLARAFRLDDAERTHLFDLASAADGTSALARPRRRSTSRWSARP
Gobs_1568       RLERGNANGVSDSVLEALARVLQLDDAERAHLYDLTRGMTAA---ARSRRRP-ARPGVRP
RO00570         RMERGNLAGASDSVLDALVSALQLDDAERDHLFALARQAQAS----TTRRPRTPLVTVRP
CgR_0165        RLERGNLSGVSEEVLAAVAGALQLDEAERSHLFDLAKATNSQ(4)RNTRGATRAQEKVRP
Consensus/80%   RLE.G.....S..VL.A.A.AL.LD..E..HL..L.......................RP

SCO4944         ALLQLLDSIDT--VPAYVSGRRSEILAWNRMAAALFG-----DWGKLPA---AERNWARL
SACE_1844       ALQQLIDAMES--TPAIVLGRRLDILGWNRLACALLG-----DFPAMEP---GRRNLARQ
FRAAL2385       SIRLLLDELRN--SPALVIGRRNDVLAWNRLGHALLCSHLPADAPQRCH---GRPNLARE
KSE_00670t      AAAQLIAAMTD--VPAVILDRRNDVLAWNPLGHALLAGHIDYTAPQSPS---ERPNLTRM
caci_7725       GLRRLLESATD--VPAYVVGLGTDVLAWNQLAAALLI-----DFGTLAP---HERNLAWL
AMED_8063       ELQHLLD-AVQ--TPAFVFGRYLDVLAWNALGGAVSF-----DFGRLE-----ERSMPKL
Micau_3909      AMHQLLASMTE--HPALILGRRTDVLASNALGRALLT-----DWTLLPP---RHRNYIRW
Amir_0367       GVHQLFDSLDG--VPALALGRRTDVLAANRLARALFT-----DFEAIPA---PRRNYARW
Msmeg_0523      VTLRLLELLDQSHSPAFVLGRRLDVLAHNRLAGALIT-----EFRELPA---PQRNQARF
Sros_3720       GLHRILDALAG-AAPAFILGRRMDVLASNRLARALIT-----DFDALPQ---RERNLARY
Aaur_2039       GVQLTLDAISG--SPAFIRNGRLDLLATNALGRALYADIYDSPAGP--------PNHARF
Krad_2718       GLQWALDAFTA--GPAIVRNGRMDLLATNTLGWAMHSSLYASLDAAGGG---THPNFARY
Gobs_1568       GVQQLLDAMTV--VPAFVQNARLDVPAANPLARALYADLFDDAEPGSAG---RTPNHARY
RO00570         AIQQVLDAITD--APAWVRNGRHDIIAMNDLARALYSPVLADPRRP--------ANTTRF
CgR_0165        EVQRILDAMAD--TPAFILTDRADMLTTNQLGRALFAPLYDSPVSPGTSGQAETVNIARF
Consensus/80%   .....L........PA.....R.D.LA.N.L..AL....................N..R.

SCO4944         VFLNPDY-RELFVDWDQKAYDMVSFLRMDAGRHPDDPRLSALVGELSVKSEEFRRLWATH
SACE_1844       VFLEPAA-REFYVDWTAKATEIVALLRLDAGRHPDDEALCALVEELSGRSEVFSRLWADR
FRAAL2385       FVLEAHA-RDLFVDWRRKVDDVVAYLRLSSGMHPDDTKLNALIGELCVKSELFATLWARH
KSE_00670t      LFLDEHT-RELHTAWEDEAKTSVAALRLTAGRHPDDRRLAELIGQLAMKSDEFAGMWSRH
caci_7725       LCHDEGF-RALFTDPVAKMQDIAAYLRLDIGRHPDDPALGTLIAELCRDAD-FAAEWARH
AMED_8063       VFTDERA-KQLHPEWDAVCRDVVANLRAERGKHPDDPHFARLAGELSVASERFRELWAEH
Micau_3909      VFLDPGA-RDAFLDWPAVAADVVGTLRLYAGRNPDDPQLSELVGELSIKSPEFRAWWNGR
Amir_0367       MFLDPAA-RALFADWEAQARNAVESLRLEVGRDPGDRATLALLAELRELSPEFDRWWEQH
```

```
Msmeg_0523      VFFDPHA-RELYADWDEVAADTVAMLRLDAGRYPDDDKLSALVGELSIRSEEFRSWWSDH
Sros_3720       MFLDEAA-RELHVDWDGAAVETVAILRLDAGRHPDDPQLAELVGELAVKSEEFRTWWADH
Aaur_2039       LFLDPRS-HDFYTDWERAANDTVAIMRTEAGRDPYDRGLSDLVGELSTRSEEFRVRWASH
Krad_2718       TFLDADA-RRFYPDWATAAATCVSILRTEAGRDPHDKVMHDLVGELSTRSEEFRALWSSH
Gobs_1568       AFLDARA-TDFYPDWNRVAADIVSQLRAEVGRSPDDRELTELIGELTTRSQHFSTLWATH
RO00570         VYLHPQAAEEFFVDYDTIARDAAAMLRLEAGRNPHDKALIALVGELSTQSELFRQRWASQ
CgR_0165        TFLDPVA-REFFPHWEHNAADLAASLRSTAGRHPHDTVFSNLIGELATRSQDFAQLWADH
Consensus/80%   .FL.........DW...A...V..LR...GR.P.D.....L.GEL...S..F...W..H


SCO4944         DVKEKSYGVKRMRHPLVGDLTLSFETFRLVDDDEQAFITYHAEPGSPSADALRLLASWGT
SACE_1844       DVRDLGYGRKQLRHPVVGPLTLAYETMHLPDPD-QKVIAYHAEPGSPSSESLNLLATSTL
FRAAL2385       PVRDCTNTVREFRHPLVGRLTLFEEVMRLP-DAGQRIVVMTAEPGSASAENLQLLASLDL
KSE_00670t      PVRSCTFGTKLLHHPFVGALELSFESMQLADDSGQRMLAYSAPAGSPSQAGLQLLAGTLL
caci_7725       DVRDKTHGSYTYRNPVVGEITLDYETFRAPDDPDQALILQTAPEGSPGEAALQLLAAWPQ
AMED_8063       AVQEKARGWKLIMNPVVGELRLRYETLRLPDDPDQGLVMYHAEPGSASERALGLLASWIA
Micau_3909      HVHERTHGTKRMHHPAVGPITLRYEALVLPGDPDQTLFLYTTDPGSPSYDNLRLLGSLAA
Amir_0367       RVHQRTHGSKRLTHPLVGELTVEYETVALPGDPDTTLFLYTTAAGTASRQALDLL---AS
Msmeg_0523      TVKQRTVGTKAYHHPLVGDLAVTYQALNPSGDPDQTLFIYTTEPGSPDETALRLLANWHE
Sros_3720       NVRERTHGTRRYHHPVVGELVLSHESVTFPGDADQTMCVYTTEPGSPSEAALHLLATWTA
Aaur_2039       NVRQHYTGKKHFRHRIVGDLHLLYEALELSADAGLTLTVYNAEPGTGTADALQLLASWAA
Krad_2718       DVRLHGAGTKRFHHTAVGELDLAYESLEMVAEPGLTLTLYAAEPASPTAQALDLLATWAA
Gobs_1568       NVRWHTTGTKRFHHPVVGDLTLAYEGMALTADRGQTLITFTAEPGSPSQQALAFLASWAT
RO00570         DVRFHRSGRKRLRHPSVGQLDLDFEALELPSEPGLQLNIYTAAAGTPSADALKLLASWAA
CgR_0165        NVRLHRVGRKTLHHPIVGDLELDFETLILPADPDQSLIVYSAAPGSDAAQNLRLLASWTV
Consensus/80%   .V.....G.K...HP.VG.L.L..E......D.......Y....GS.....L.LLA....


SCO4944         DAAASLPA---------------------
SACE_1844       VPAG-------------------------
FRAAL2385       TAAAPGPQRLPADPAPGGGPGTGGS(16)
KSE_00670t      SHPPHG-----ARPAARG--HLAGE(5)-
caci_7725       TVTAS-STAP-------------------
AMED_8063       EAPLTRANFPSL-----------------
Micau_3909      RTAPAPGRASSTPLPDASRTEPNPS----
Amir_0367       WTLTALAR---------------------
Msmeg_0523      GGEQGSRAASARKSSEA------------
Sros_3720       PHPPVTALRQDGQGREE------------
Aaur_2039       TALTIPEDSGAYRDISNTSSRSDRG----
Krad_2718       TER-LTHDASSRVDRSSTP----------
Gobs_1568       SPTQRAAQDRANEDHTT------------
RO00570         SQDQLGTEHDAPSRPH-------------
CgR_0165        TGTDKTGTDRAFHPADDVPSLEE------
Consensus/80%   .............................
```
