# Supplementary material for: Structure of an MmyB-Like Regulator from C. aurantiacus, Member of a New Transcription Factor Family Linked to Antibiotic Metabolism in Actinomycetes
Source: PLoS One. 2012 Jul 26;7(7):e41359. doi: 10.1371/journal.pone.0041359 (PMC3406030; doi:10.1371/journal.pone.0041359)
Supplement: Table S1 — Members of the MmyB family Xre-type regulators found in S. coelicolor A3(2). (PDF) [file pone.0041359.s003.pdf]

Paralog	Divergently transcribed gene	Intergenic region bp	Predicted function of gene product(s)	Comment
**Family 1**				
mmyB	*mmyY*	230	Nuclear transport factor 2-like protein	Methylenomycin biosynthesis
SCO0110	*SCO0111*	187	3-ketoacyl-(acyl-carrier-protein) reductase	
SCO0233	*SCO0234-SCO0235*	289	Both genes encode an SDR	
SCO0236	*SCO0237*	200	SDR	
SCO0307	*SCO0306*	123	Possible pseudogene	SCO0311 PKS; SCO0312 acyl-CoA reductase
SCO0891	*SCO0890*	102	SDR	
SCO2501	*SCO2502*	111	Similar to actinorhodin exporter ActII-2	
SCO2537	*SCO2537*	107	Similar to cephamycin transporter CmcT from *Nocardia lactamdurans*	Fused to SCO2538
SCO4680	*SCO4681-SCO4682*	45	SDR, downstream gene encodes 4-oxalocrotonate tautomerase	
SCO4944	*SCO4945*	169	MDR (alcohol dehydrogenase)	Highly conserved^
SCO6926	*SCO6925*	582	Membrane protein; gene contains TTA codon	Possibly controls lantibiotic cluster *SCO6927-6930*
SCO7140	*SCO7141*[#]	28	SDR	
SCO7706	*SCO7707*	138	Atu4866 family protein	SCO7705 similar to tetracyclin resistance oxidoreductase
SCO7767	*SCO7768-SCO7769*	130	Nuclear transport factor 2-like protein, similar to RANGDP-binding; fused to gene for GDP-sugar epimerase	Next to *SCO7766,* homolog of *actVA4*
SCO7817	*SCO7818*	117	SDR (2,3-dihydro-2,3-dihydroxybenzoate dehydrogenase)	
Family 2				
SCO6539	*SCO6540-SCO6541*	90	DCoH domain protein; downstream gene similar to *S. purpurascens rdmD* (anthracycline biosynthesis)	Highly conserved in *Streptomyces*

[#] no shared promoter region.

^ next to ortholog of *SCO4945* in all *Streptomyces* sp, *Rhodococcus* sp, *Saccharopolyspora erythraea*, *Kineococcus radiotolerans, Arthrobacter aurescens*.
